# In Vitro liposome release profile prediction using explainable machine learning approaches

**DOI:** 10.1371/journal.pone.0354293

**Published:** 2026-08-11

**Authors:** Hamza Abu Owida, Sameer Ahmad Hasan, Areen Arabiat, Suhaila Abuowaida

**Affiliations:** 1 Department of Medical Engineering, Faculty of Engineering, Al-Ahliyya Amman University, Amman, Jordan; 2 Department of Biomedical Engineering, School of Applied Medical Sciences, German Jordanian University, Amman, Jordan; 3 Department of Communications and Computer Engineering, Faculty of Engineering, Al-Ahliyya Amman University, Amman, Jordan; 4 Department of Data Science and Artificial Intelligence, Faculty of Prince Al-Hussein Bin Abdallah II for Information Technology, Al Al-Bayt University, Mafraq, Jordan; National University of Rosario, ARGENTINA

## Abstract

Formulation features and test settings influence liposomal in vitro release (IVR) profiles, yet it is challenging to examine these multivariable associations across varied literature data. We created an explainable computational workflow in this proof-of-concept study for classifying liposomal release phenotypes and identify formulation/assay features linked to slow and fast release. Benchmarking kinetic models, simulating Weibull-parameterized release curves on a shared 0–168 h grid, clustering profiles using PCA and k-means, and training supervised classifiers on formulation and IVR descriptors were all done using a publicly available Accelerated IVR dataset. Slow, moderate, and fast kinetic phenotypes were found using PCA-k-means; 98.4% of the variance was explained by the first two principal components. XGBoost demonstrated the best cross-validated performance across evaluated models for the extreme slow-versus-fast subgroup (n = 59); however, class-wise recall and precision indicate preliminary rather than conclusive prediction performance. The most informative descriptors identified by feature selection and SHAP interpretation were media pH, drug loading, weighted lipid transition temperature, and media temperature. These results indicated that risk-based IVR technique development and hypothesis creation for liposomal formulations can be supported by explainable ML; nevertheless, prior to translational or regulatory usage, prospective validation on independently generated datasets is required.

## 1. Introduction

Liposome formulations have been shown to have varying levels of in vitro release (IVR) under different experimental conditions (formulation characteristics, e.g., lipid composition, drug concentration and amount, vesicle diameter and size distribution) and assay conditions (temperature, pH, medium composition and hydrodynamics). This dependence complicates quantitative comparison across studies and motivates predictive characterization tools that jointly

However,phospholipid vesicles, which were initially identified as therapeutic nanoparticle-based delivery methods for both hydrophilic and hydrophobic active medicinal substances, gave rise to liposomes [1,2]. Liposomes have been widely used to improve the apparent solubility of poorly soluble medications, reduce systemic toxicity by altering the biodistribution of pharmaceuticals, and enable either ligand-directed or passive targeting to targeted tissues [2–4]. Lipid carrier systems can significantly reduce the dose-limiting toxicities associated with free medication while protecting therapeutic exposure, as shown by the clinical effectiveness of long-circulating (“stealth”) liposomes [5–7]. These advantages have aided in the commercialization of a number of liposome-based medicines, such as doxorubicin formulations, which have been often mentioned as instances of successful regulatory approval of nanomedicine products. [4,7]. At the same time, the performance of liposome products is highly dependent upon the physical chemical characteristics of the system and its release behavior, which supports the broader regulatory focus on the establishment of comprehensive characterization and control processes for complex drug products [8,9].

As a result, in vitro release-dissolution testing will be crucial for these kinds of products’ development and quality control. The use of dissolving to evaluate batch-to-batch quality and verify process/formulation modifications made during development is emphasized in regulatory guidelines for immediate-release oral medicines [10]. Pharmacopeial guidelines for the in vitro release of drug products provide a thorough explanation of method development and validation elements like resilience to agitation, medium composition, sampling design, and sink settings [11]. Furthermore, comparison metrics like the similarity factor f2 are becoming more widely used to determine whether modifications to a manufacturing process or formulation lead to significant changes in the dissolution profiles [12]. For complicated pharmaceutical drugs, however, the measurement procedure itself may be a significant source of variability. For instance, there are presently no comprehensive in vitro release techniques available for liposome assessment. Furthermore, depending on the chosen lipid membrane, hydrodynamic conditions, and the capacity to maintain sink conditions, the different methods that have been reported for the release testing of liposomes, such as dialysis variations, sample-and-separate, continuous flow, in situ methods, and modified USP apparatuses, can produce different apparent release kinetics [9]. The “release profile” is an emergent property of the combination of the formulation composition, the physio-chemical characteristics of the formulation, and the experimental conditions under which it was tested. This illustrates a larger issue for the field as a whole.

Numerous key material attributes (CMAs) and critical quality attributes (CQAs) interact to affect medication release and stability [13–15]. CQAs for lipid nanomedicines, like liposomes, usually include the vesicles’ mean diameter and polydispersity index, zeta potential, drug loading, and encapsulation efficiency, all of which might affect the drug’s stability and release [2,16]. The observed kinetics can also be influenced by the parameters of in vitro release (IVR) techniques, including temperature, pH, medium composition and surfactants, agitation/hydrodynamics, and equipment design [9]– [11]. Standardized procedures and mathematical models that quantitatively relate the characteristics of the formulation and IVR conditions to the release kinetics of drugs from complex systems where multiple mechanisms of release, such as diffusion, erosion, phase transformation, and precipitation, may occur simultaneously are still lacking, despite the acknowledged impact of these factors [9].

By identifying non-linear associations from multivariate datasets and creating predictive models that can be examined for feature importance, machine learning (ML) provides a workable solution to close this gap. ML has been used in pharmaceutical development to predict tablet dissolution/release profiles using spectroscopic and process data, allowing for surrogate or real-time release testing strategies [17,18]. More recent research has shown kinetic-parameter prediction and profile-level prediction using ensemble models like gradient boosting [19]. ML has also been used for other controlled-release platforms, such as polymeric microspheres, where time-dependent release behavior is simultaneously determined by formulation and process factors [20–23]. With increasing interest in ML-guided lipid nanoparticle and liposome design, machine learning (ML) has been applied in lipid-based formulation science to predict and optimize nanoparticle features (e.g., size, PDI, zeta potential, encapsulation) and to justify formulation factor relevance [16,24]. Nevertheless, applications that explicitly model IVR profiles for complex nanomedicines are still relatively rare, and systematic, quantitative linking of formulation and IVR factors to release behavior has not been widely established [9,16]. This is in contrast to the rapid expansion of ML for property prediction and for traditional dissolution tasks.

The present work introduces an integrated, explainable workflow for liposomal IVR analysis that benchmarks common kinetic models, standardizes heterogeneous release profiles via Weibull-based simulation on a common time grid, assigns kinetic phenotypes (slow/intermediate/fast) using unsupervised learning, and predicts kinetic class from formulation and IVR descriptors using supervised machine learning. This framework is designed to support the formulation screening process, comparative evaluation of IVR methods, and risk-based decision making for liposomal formulations by enabling a common framework for summarizing, clustering and modeling liposomal drug product release behavior across diverse experimental conditions.Relation to Yanes et al.‘s work: Yanes and associates described a machine-learning approach for categorizing release-profile kinds from formulation, lipid-property, drug-type, and IVR-method descriptors [24,25]. They also constructed the underlying AI-ready liposome IVR resource. Although the current document is in line with that goal, its emphasis is different in three respects. In order to remove ambiguity from intermediate profiles, it first concentrates on an interpretable slow-versus-fast extreme-kinetics classification task using the public Accelerated IVR dataset as a secondary, repeatable data source. Second, it integrates PCA-k-means phenotyping, greedy feature elimination, Weibull simulation on a standardized 0-168 h grid, f2-based benchmarking, and TreeSHAP interpretation into a unified workflow. Third, by connecting temperature, lipid transition temperature, drug loading, and pH to likely liposomal release processes, the current analysis frames the results for pharmacological interpretation. Therefore, rather than taking the place of Yanes et al.’s core database-development work, the study should be viewed as a supplemental, secondary explainable-ML analysis.

## 2. Methods

### 2.1. Study design and data sources

The Accelerated IVR public dataset and accompanying coding, which are housed in a GitHub repository with a CC-BY license, were utilized in all studies [26]. An SQLite relational database with linked tables for article provenance, formulation composition, lipid and excipient identities, physicochemical descriptors, IVR assay conditions, and digitized cumulative release profiles was initially assembled from 34 scholarly publications. WebPlotDigitizer (v4.6) was used to digitize drug-release curves. In practice, each release profile can be linked to its source article, formulation, lipid components, assay settings, and computerized time-release data without duplicating or combining records thanks to the records being stored across relational tables. The precise variables required for modeling may be retrieved via structured SQL queries according to this framework, which also guarantees provenance.

This work adds a Supplementary Table S1 review and clearly indicates the Accelerated IVR article and repository as the source of the curated database according to the reader’s concern regarding the 34 source publications. Before being resubmitted, Supplementary Table S1 should identify all 34 primary source articles precisely as they are listed in the public database information. This would allow readers to verify each source record that was utilized to create the dataset.

In order to benchmark kinetic models of cumulative liposomal drug release, create standardized release curves from Weibull parameterizations, use unsupervised learning to identify kinetic phenotypes, and create supervised models that predict kinetic class from formulation and IVR characteristics, this study developed an artificial computational workflow.

Table 1 summarizes each dataset’s composition and usage (release-profile cohort, metadata-annotated cohort, cleaned metadata table, and kinetic-extremes subset). In summary, 169 Weibull-parameterized release profiles for kinetic-model benchmarking and PCA-k-means clustering had been included in the release-profile cohort; 78 formulation/IVR records with three-kinetic classes were utilized in the metadata-annotated cohort; 77 unique formulation-assay records were retained in the cleaned metadata table after one duplicate entry was excluded; and 59 records corresponding to the slow (n = 34) and fast (n = 25) classes used for binary supervised learning

**Table 1:** Study datasets and analysis flow.

Dataset	N	Contents/ purpose	Used in
Release-profile cohort	169	Weibull-parameterized release profiles; simulated on a 0–168 h grid	Kinetic-model benchmarking; PCA–k-means clustering
Metadata-annotated cohort	78	Formulation and IVR descriptors with a three-class kinetic phenotype label	Descriptive statistics; candidate feature set
Cleaned metadata table	77	Metadata cohort after removing one duplicate formulation–assay record	Model comparison; feature selection
Kinetic-extremes subset	59	Binary subset: slow (34) versus fast (25) phenotypes	Supervised classification; SHAP; permutation test

### 2.2. Feature definitions and preprocessing

Table 2 summarizes possible predictors for the cleaned metadata table (n = 77), including data types and units, preprocessing methods, encoding strategies per model family, and variable-level missingness. Missingness is reported prior to within-fold imputation.

**Table 2:** Possible predictors for the cleaned metadata.

Predictor	Type	Unit	Transformation	Encoding (by model family)	Imputation	Missing (n/%)
IVR temperature	Continuous	°C	None	Numeric for all models	Median (within-fold)	0 (0.0%)
IVR medium pH	Continuous	—	None	Numeric for all models	Median (within-fold)	0 (0.0%)
Drug loading	Continuous	% (w/w) or reported unit	None	Numeric for all models	Median (within-fold)	0 (0.0%)
Hydrodynamic diameter (Z)	Continuous	nm	log10(Z)	Numeric for all models	Median (within-fold)	0 (0.0%)
Weighted lipid transition temperature (Tm,weighted)	Continuous	°C	None	Numeric for all models	Median (within-fold)	0 (0.0%)
API grouping	Categorical	—	None	One-hot (linear/SVM/kNN); ordinal integer (tree/boosting)	Mode (within-fold)	0 (0.0%)
Assay format (if present)	Categorical	—	None	One-hot (linear/SVM/kNN); ordinal integer (tree/boosting)	Mode (within-fold)	0 (0.0%)
Liposome structural class (if present)	Categorical	—	None	One-hot (linear/SVM/kNN); ordinal integer (tree/boosting)	Mode (within-fold)	0 (0.0%)

As listed in Table 2, potential predictors were IVR medium pH, IVR temperature, drug loading, hydrodynamic diameter, lipid-mixture descriptors, API grouping, and assay-format descriptors. Hydrodynamic size was log10-transformed because it comprised several orders of magnitude. The composition-weighted mean of the phase-transition temperatures of the lipid components in a formulation was used to calculate the weighted lipid transition temperature (Tm,weighted): Tm,weighted = Σ(wi × Tm,i), where wi is the reported molar or mass fraction of lipid i and Tm,i is its literature transition temperature. Molar fractions were given priority where precise molar fractions were provided; otherwise, stated composition fractions were consistently employed throughout the formulation record. One-hot encoding is the process of turning a category variable—like an assay format or API group—into distinct binary indicator columns. For instance, linear, SVM, and k-nearest-neighbor models can employ categorical data without imposing an arbitrary numerical order when three API groups are converted to three columns coded as 0 or 1.


$$\begin{array}{c} {\text{Z}}_{\text{log}}\;=\;{\text{log}}_{\text{10}}(\text{Z}) \end{array}$$ (1)

Furthermore, the cleaned metadata database was used to quantify missing values, which are summarized per variable in database 2. The imputation model fitted on the training fold was then applied to the held-out fold. For modeling, missing values were imputed within each training fold using the mode for categorical variables and the median for continuous variables. The predictors indicated in database 2 had no missing values after curation in the cleaned metadata database utilized here, hence each retained predictor’s reported missingness was (0.0%).

### 2.3. Kinetic models and f_2_ similarity analysis

The parameters and notation for the kinetic model are as follows: Q(t) is the cumulative percentage of medication released at time t (hours), restricted to the interval [0, 100] across the fitted time range. K0 (zero-order rate), k1 (first-order rate constant), kH (Higuchi constant), kKP and n (Korsmeyer–Peppas scale parameter and exponent), kHC (Hixson–Crowell constant), and α and β (Weibull scale and shape parameters, respectively) are the fitted parameters. Through Equations (3) to (8), the model expressions listed below were used in the benchmarking procedure as shown in Table 3.

**Table 3:** Kinetic model expressions for cumulative release Q(t).

Kinetic model	Model expression
Zero-order:	Q(t) = k0 t
First-order:	Q(t) = 100 [1 – exp(-k1 t)]
Higuchi:	Q(t) = kH t^(1/2)
Korsmeyer-Peppas:	Q(t) = kKP t^n
Hixson-Crowell:	Q(t) = 100 [1 – (1 - kHC t)^3]
Weibull:	Q(t) = 100 [1 – exp(-(t/ alpha)^beta)]

Pharmaceutical interpretation of the kinetic models: zero-order release is frequently employed as an idealized sustained-release benchmark, assuming a constant release rate throughout time. First-order release makes the assumption that when the unreleased drug fraction drops, correspondingly does the release rate. The Higuchi model predicts release proportionate to the square root of time and illustrates diffusion-controlled release from a matrix-like structure. The Korsmeyer-Peppas model is an empirical power-law model whose exponent n, depending on system shape, gives a qualitative indication of whether release is more in line with anomalous transport, Fickian diffusion, or relaxation/erosion-controlled behavior. The Hixson-Crowell model explains how variations in particle or dosage-form surface area during erosion or dissolution impact release. Even when a single physical process is not assumed, the Weibull model—a versatile empirical model in which the shape parameter controls curve curvature and the scale parameter controls typical release time—is helpful in assessing varied release patterns. The model forms used for benchmarking were shown in Table 3:

For each profile, agreement between the observed release curve and the model-predicted curve at matched timepoints was quantified using the similarity factor f2, with higher values indicating closer similarity as shown in Equation (9):


$$\begin{array}{c} {\text{f}}_{\text{2}}=\text{5}0{log}_{\text{1}\text{0}}{\left\{\text{1}+\frac{\text{1}}{n}\sum_{t=\text{1}}^{n}{\left(R_{t}-T_{t}\right)}^{\text{2}}\right\}}^{\frac{-\text{1}}{\text{2}}}\text{1}\text{0}\text{0} \end{array}$$ (2)

### 2.4. Weibull simulation and kinetic descriptors

Each parameterized release curve was simulated on a homogeneous grid from 0 to 168 h in 1 h increments to allow for direct multivariate comparison between profiles. Equation (10) shows how a kinetic half-time descriptor, which is the time to 50% release (t_20_), was obtained from Weibull parameters and utilized as an interpretable ordering measure for cluster recognizing.


$$\begin{array}{c} {\text{t}}_{\text{50}}=\alpha (\text{In }\text{2}{)}^{\frac{\text{1}}{\beta }} \end{array}$$ (10)

### 2.5. Unsupervised phenotyping by PCA and k-means clustering

In order to prevent dominance by late-time saturation values, simulated release vectors were merged into a matrix of profiles by timepoints and normalized by z-scoring each timepoint across profiles. Then, using repeated random initializations and a set random seed for repeatability, means clustering was carried out in PC score space with k = 3 clusters using principal component analysis (PCA) to provide low-dimensional PC scores capturing greatest variance in release behavior. Cluster-average release curves were calculated as the mean simulated release at each timepoint inside a cluster, and clusters were classified as slow, moderate, or fast by increasing median t•¹.

### 2.6. Supervised machine-learning workflow

The kinetic-extremes subset (n = 59) was used to train binary classification models to differentiate between slow and fast kinetics. Logistic regression, k-nearest neighbors, RBF-kernel support vector machines, naïve Bayes, random forest, gradient boosting, and XGBoost were among the potential techniques. Stratified five-fold cross-validation was used to evaluate the model. The main metric utilized to define performance was balanced accuracy, which was supplemented by the F1 score and the Matthews correlation coefficient (MCC) to evaluate performance under class imbalance. On the other hand, in order to account for the slow/fast class ratio, XGBoost models were trained using a logistic objective and class weighting. Randomized search inside cross-validation was used to tune hyperparameters, and balanced accuracy served as the model selection factor.

The algorithms are explained simply as follows: logistic regression estimates a weighted linear boundary between slow and fast profiles; k-nearest neighbors classifies a sample based on the most similar neighboring formulations; the RBF-kernel support vector machine creates a nonlinear boundary in transformed feature space; naive Bayes applies probability rules under simplifying independence assumptions; random forest averages multiple decision trees to minimize overfitting; gradient boosting builds trees sequentially so that each tree corrects errors from earlier trees; and XGBoost is an optimized gradient-boosted tree implementation that can capture nonlinear interactions while limiting overfitting through normalization. Only unsupervised profile representation was represented by PCA; release phenotypes were assigned using k-means; feature contributions to model predictions were interpreted using SHAP; and if the observed balanced accuracy outperformed chance performance was assessed using permutation testing.

Methodical validation procedure: (1) Create five stratified folds from the kinetic-extremes data. (2) Only apply preprocessing (encoding, log-transform, and imputation as shown in Table 2) to each fold’s 4/5 training portion. (3) During the training phase, perform a randomized hyperparameter evaluation for each candidate model using an inner cross-validation loop; select hyperparameters that maximize mean balanced accuracy. (4) Throughout the training phase, refit the model using the selected hyperparameters, then evaluate it using the held-out fold. (5) By integrating performance across folds, report the mean ± standard deviation for balanced accuracy, F1 score, and MCC.

### 2.7. Feature selection, interpretability, and statistical testing

Greedy backward elimination was used with XGBoost to find efficient predictions. We started with the entire feature set and eliminated one feature at a time, choosing the deletion that yielded the highest mean balanced accuracy after assessing each single-feature deletion under stratified five-fold cross-validation. This procedure establishes a path of elimination. In order to prioritize parsimony without compromising predictive performance, the smallest set along this path whose mean balanced accuracy matched the maximum observed (within cross-validation uncertainty) was selected as the final feature subset.

TreeSHAP values calculated from the trained XGBoost model were used to evaluate the interpretability of the model. The distribution of per-sample SHAP values and their correlation with slow or rapid predictions were shown using beeswarm plots, and features were sorted by mean absolute SHAP value. A permutation test was used to determine whether classifier performance is superior to chance. The test statistic was the average balanced accuracy in stratified three-fold cross validation. The model was fitted again after randomly permuting each label 100 times to create a null distribution of balanced accuracies. To roughly balance computational time and p-value resolution, we used 100 permutations and calculated a one-sided p-value as the percentage of permuted balanced accuracies that are at least as great as the observed balanced accuracy (the minimum achievable one-sided p value is approximately.0099 using the + 1 correction).

### 2.8. Software and reproducibility

All analyses were conducted in Python 3 using NumPy, pandas, SciPy, scikit-learn (for PCA, k-means clustering, cross-validation, and baseline models), XGBoost (for gradient-boosted trees), SHAP (for TreeSHAP explainability), and Matplotlib (for visualization). Random seeds were fixed when necessary for stochastic components such as k-means initialization and model fitting. Preprocessing procedures that depend on data distributions, including imputation and categorical encoding, were performed inside cross-validation folds to prevent information leakage.

## 3. Results

### 3.1. Dataset composition and scope

169 Weibull-parameterized release profiles covering quick to sustained release behaviors over a standardized 0–168 h timeframe comprised the release-profile cohort. Three kinetic phenotypes (slow, n = 34; intermediate, n = 19; fast, n = 25) comprised the 78 labeled occurrences in the metadata-annotated cohort. The kinetic extremes were the focus of supervised modeling, which kept 59 records (slow, n = 34; fast, n = 25).

### 3.2. Kinetic model benchmarking using f_2_

**Fig 1:**
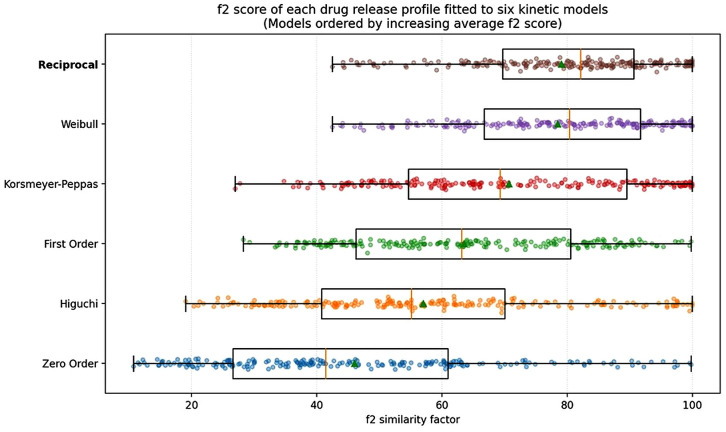
Average f_2_ similarity factor across kinetic models. Horizontal boxplots summarize f_2_ values comparing digitized release profiles with model-predicted profiles at matched time points. Points represent individual profiles, boxes indicate interquartile ranges, whiskers extend to 1.5 x IQR, and annotated mean values support direct comparison among kinetic models.

Significant differences in similarity between various kinetic models were found through benchmarking based on the f₂ similarity factor. Fig 1. Shows shows how similar the digitized release profiles are to the model-predicted release profiles across different kinetic models, using the f2f_2f2 similarity factor. In this figure, the horizontal boxplots summarize the distribution of f2f_2f2 values calculated at matched time points between the experimental (digitized) and predicted profiles. Each dot corresponds to an individual comparison between digitized and model-predicted profiles. The box highlights the interquartile range (from the 25th to the 75th percentile of the f2f_2f2 values), indicating the middle spread of similarity across replicates. The whiskers extend to values up to 1.5 times the interquartile range, showing the typical overall range of f2f_2f2 values. Finally, the annotated mean values allow for straightforward comparison of average similarity across the kinetic models.However, horizontal boxplots summarize the distribution of f_2_ values computed between observed release profiles and model-predicted profiles for each kinetic model; individual points represent single profiles; boxes indicate interquartile ranges; whiskers extend to 1.5 × IQR; mean f₂ values are indicated and annotated above each model to facilitate direct comparison of overall similarity across modeling choices. Because Weibull parameterized profiles functioned as the basis for the model’s development, the Weibull model was the most similar to the reference curves. First order and Korsmeyer-Peppas models outperformed the linear forms (zero order and Hixson-Crowell) in terms of f_2_ scores. As a result, many of these release profiles seem to have a non-linear curvature, which could be a sign of either a variety of release processes or mechanisms that change with time.

### 3.3. Weibull-simulated profiles and kinetic heterogeneity

**Fig 2:**
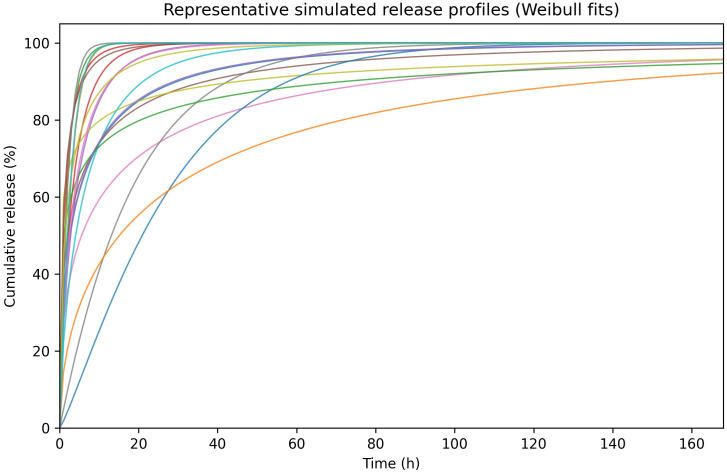
Simulated drug-release profiles generated from fitted Weibull parameters. Representative cumulative release curves were reconstructed from fitted Weibull alpha and beta parameters on a standardized 0-168 h time grid. Each colored curve represents an individual liposomal formulation release trajectory generated from Weibull parameterization.

Simulation of cumulative release using fitted Weibull parameters produced fixed-length profiles that preserved broad heterogeneity in kinetic behavior The simulated curves spanned rapid early release with near-saturation at short times to sustained, delayed release over the full 168 h window, supporting their use as standardized inputs for multivariate analysis. However, Fig 2 depicts Simulated drug release profiles generated from fitted Weibull parameters. Representative cumulative release curves simulated from fitted Weibull α (scale) and β (shape) parameters on a uniform 0–168 h time grid (1 h increments). The figure illustrates the range of kinetic behaviors captured by the parameterization, including rapid, intermediate, and sustained release trajectories.

### 3.4. PCA–k-means clustering resolves kinetic phenotypes

**Fig 3:**
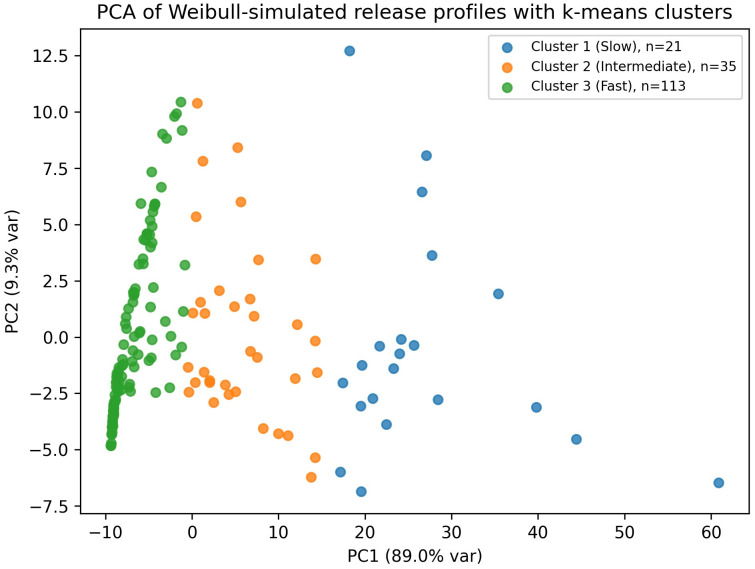
PCA of standardized simulated Weibull profiles with k-means cluster assignments. Each point represents one release profile projected onto the first two principal components and colored by k-means cluster membership. The plot shows separation of slow, intermediate, and fast kinetic phenotypes.

**Fig 4:**
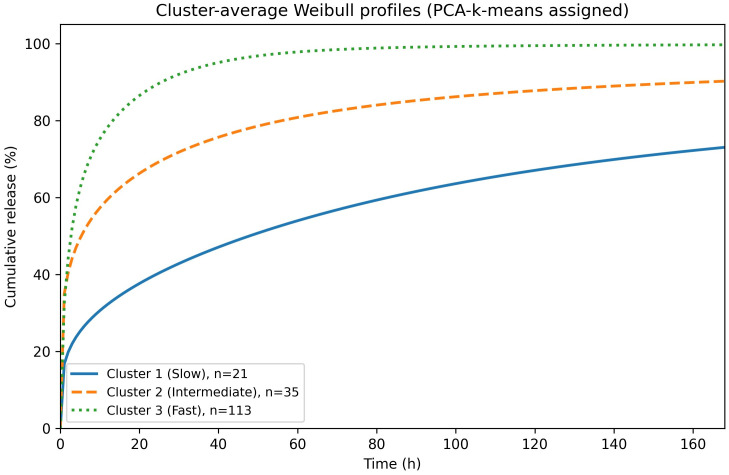
Cluster-average simulated release profiles assigned by PCA-k-means clustering. Curves show the mean cumulative release trajectory for each cluster over the standardized 0-168 h grid, supporting the interpretation of the clusters as distinct kinetic phenotypes.

The PCA of standardization of simulated release vectors resulted in a lower dimensional representation which retained most of the variation of the release behaviors. For this data set, PC1 and PC2 represented 98.4 percent of the total variation present in the standardized simulated release vectors that were clustered to identify kinetic phenotypes using k-means (K = 3) in the PC space. The clusters identified as slow, intermediate and fast release types based on median t50 values and average release curves from each cluster were consistently separated at all time points within the 0–168 hour release period.Median t50 value for the entire data set was 2.78 hours, indicating that there is primarily rapid release behavior occurring under accelerated release testing conditions. However, Fig 3 depicts PCA of standardized simulated Weibull profiles with k-means cluster assignments. Scatter plot of PC1 versus PC2 scores derived from PCA applied to z-scored simulated release vectors. Each point corresponds to a single release profile and is colored by k-means cluster membership (k = 3). The plot highlights the separation of kinetic phenotypes in reduced-dimensional space and provides the basis for cluster assignment while Fig 4 depicts the cluster-average simulated release profiles assigned by PCA–KMC. Mean cumulative release trajectories for the slow, intermediate, and fast clusters obtained by k-means clustering in PCA space. Curves are shown on the standardized 0–168 h grid, emphasizing distinct kinetic signatures across clusters and supporting the interpretation of clusters as phenotypically meaningful release regimes.

The unsupervised phenotypes provided the label structure for downstream supervised modeling; subsequent classifier development therefore focused only on the slow and fast extreme-kinetics subset to minimize label ambiguity in the prediction task.

### 3.5. Feature selection and explainable predictors of slow versus fast kinetics

**Fig 5:**
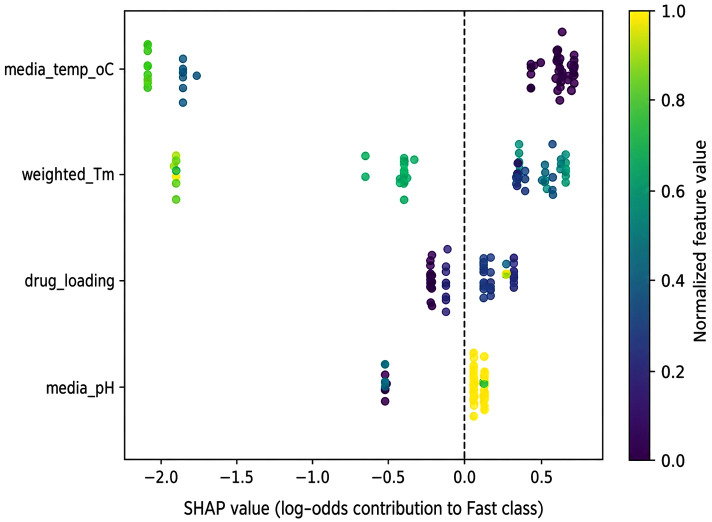
SHAP beeswarm plot showing feature contributions toward the fast kinetic class in the XGBoost model. Each point represents one formulation record; color indicates feature value, and positive SHAP values indicate increased contribution toward fast-release prediction.

**Fig 6:**
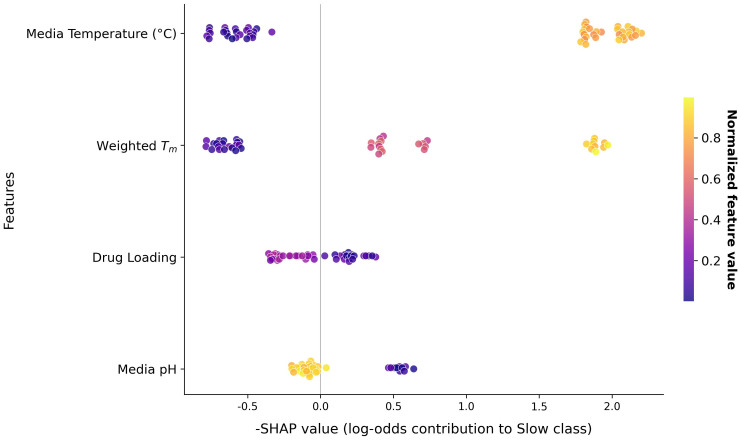
SHAP beeswarm plot showing feature contributions toward the slow kinetic class in the XGBoost model. Each point represents one formulation record; negative SHAP values indicate increased contribution toward slow-release prediction, highlighting feature regions associated with sustained release.

Greedy backward elimination was able to narrow down a number of predictive factors to a small group of variables that resulted in the maximum cross-validation of the XGBoost classifier for the two extreme kinetic classes (slow vs. fast). The results from TreeSHAP revealed that IVR temperature and the weighted lipid transition temperature were the major contributors to discrimination among these two classes; however, drug loading and the pH of the medium provided some additional information for discrimination among different formulations. The results of SHAP beeswarm plots shown in Fig 5 that shows the SHAP beeswarm plot showing feature contributions toward the fast kinetic class (XGBoost). Beeswarm plot of per-sample SHAP values for each feature in the final XGBoost classifier. Each point represents one formulation; color encodes the feature value. Positive SHAP values indicate contributions increasing the probability of fast-class prediction, enabling identification of feature-value regimes associated with rapid release and Fig 6 that depicts SHAP beeswarm plot showing feature contributions toward the slow kinetic class (XGBoost). Beeswarm plot of per-sample SHAP values highlighting feature contributions that shift predictions toward slow kinetics. Negative SHAP values indicate support for sustained-release classification and illustrate how distinct regions of feature space are associated with slow-release behavior. Both figures illustrated heterogeneous, non-linear effects for each of the predictive features across different formulations; as such, the results are consistent with the notion that there is an interaction-driven relationship between formulation properties and the operating conditions of the IVR.

### 3.6. Classifier significance and comparative model performance

**Fig 7:**
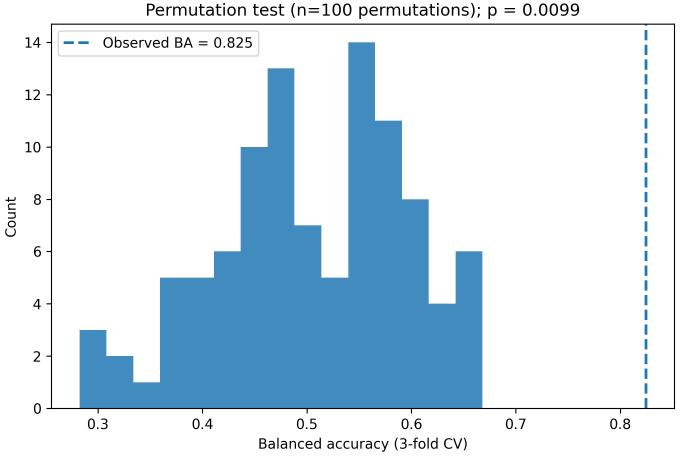
Permutation test for statistical significance of classifier performance. The histogram shows balanced accuracy values obtained after label permutation, and the vertical reference line indicates the observed balanced accuracy. The one-sided p-value was computed as the proportion of permuted scores at least as high as the observed score.

**Fig 8:**
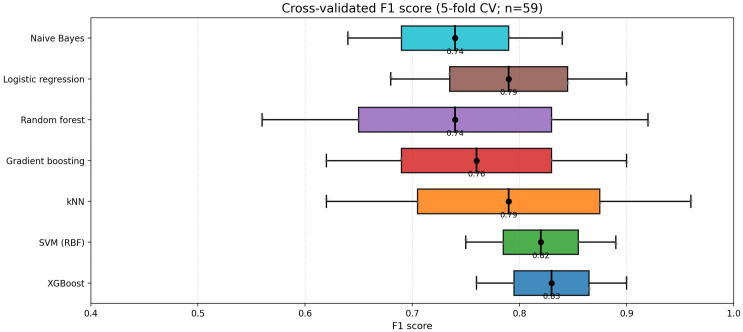
Model comparison by cross-validated F1 score. Mean F1 scores across surveyed classifiers are shown with fold-to-fold variability, allowing comparison of XGBoost with baseline approaches.

**Fig 9:**
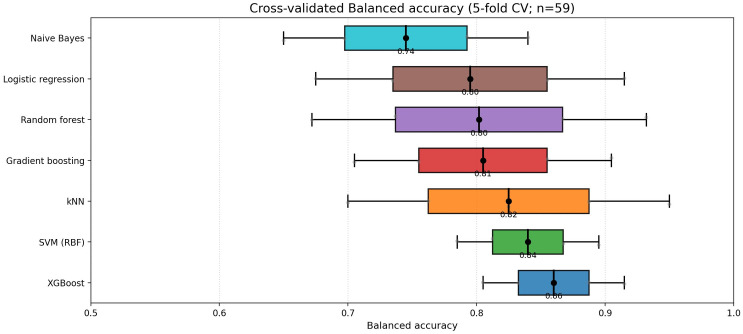
Model comparison by cross-validated balanced accuracy. Balanced accuracy was used as the primary metric to account for class imbalance between slow and fast kinetic classes.

**Fig 10:**
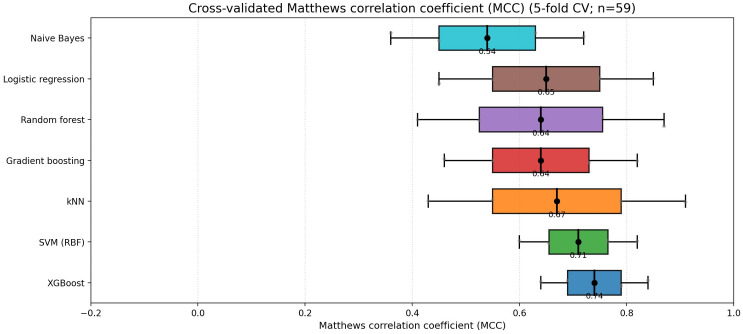
Model comparison by cross-validated Matthews correlation coefficient (MCC). MCC provides a correlation-based summary of binary classification quality under class imbalance and complements F1 score and balanced accuracy.

The permutation test confirmed that the observed classifier performance was greater than expected by chance. The observed balanced accuracy was 0.825, whereas the null distribution generated from permuted labels was centered near chance performance; the resulting one-sided p-value was 0.0099 as shown in Fig 7 that depicts whether the classifier’s performance is statistically significant using a permutation test. After randomly permuting the class labels, the classifier is run many times to generate a null distribution of performance, summarized here by a histogram of balanced accuracy values.The vertical reference line marks the observed balanced accuracy obtained using the correct (non-permuted) labels. To quantify significance, a one-sided p-value is calculated as the fraction of permutation-based balanced accuracy scores that are greater than or equal to the observed score. A small p-value indicates that the observed performance is unlikely to occur by chance under label permutation.. However,histogram of balanced accuracy values obtained under the null distribution generated by permuting slow/fast labels and refitting the classifier under stratified cross-validation. The observed balanced accuracy is shown as a vertical reference line, and the p-value is computed as the proportion of permuted scores at least as high as the observed score.XGBoost provided the strongest performance across all surveyed models under stratified cross-validation. Specifically, XGBoost achieved a mean balanced accuracy of 0.858 + /- 0.058, a mean F1 score of 0.834 + /- 0.067, and a mean MCC of 0.735 + /- 0.094, indicating robust discrimination between the slow and fast kinetic extremes. Fig 8 compares different predictive models using cross-validated F1 score. For each surveyed classifier, the figure reports the mean F1 score across cross-validation folds, and also reflects fold-to-fold variability, showing how performance changes from fold to fold.This setup makes it possible to assess whether XGBoost performs better or worse than simpler baseline methods by comparing their average F1 scores alongside the variability across folds.

while Fig 9 compares models using cross-validated balanced accuracy. Balanced accuracy is reported from performance across validation folds and serves as the primary metric because it accounts for potential class imbalance between the slow and fast kinetic classes, giving a more equitable measure than raw accuracy when one class is underrepresented and Fig 10 shows Fig 10. shows model comparison by cross-validated Matthews correlation coefficient (MCC). Mean MCC values across surveyed classifiers. MCC provides a correlation-based summary of classification quality under imbalance and complements F1 and balanced accuracy for model selection and reporting.

**Fig 11:**
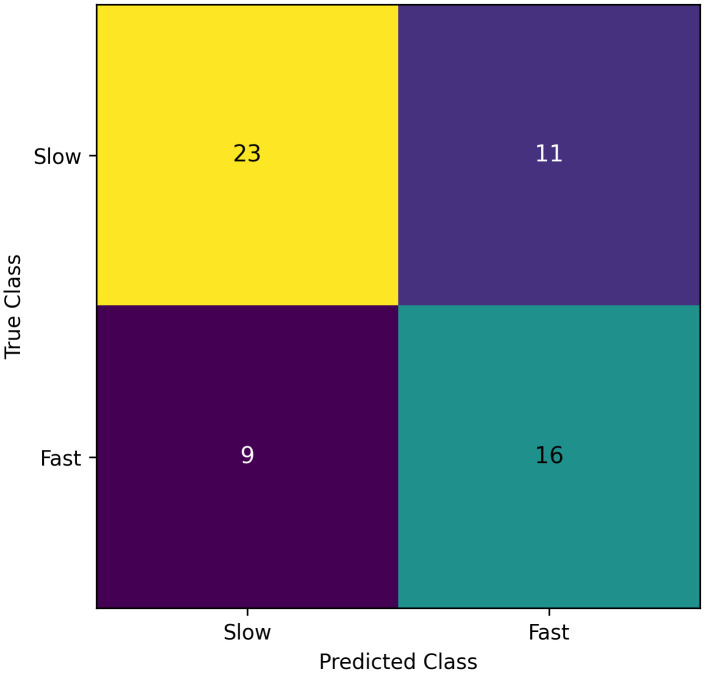
Cross-validated confusion matrix and class-wise performance for the XGBoost extreme-kinetics classifier. The figure reports true and predicted slow/fast classifications and supports cautious interpretation of class-wise recall, specificity, and precision.

Fig 11 shows the cross-validated confusion matrix and class-wise performance for the XGBoost classifier trained to identify extreme kinetics (slow vs. fast). The confusion matrix summarizes, for each fold, the counts of true slow/fast samples versus their predicted labels, making it clear where the model succeeds and where it misclassifies. The accompanying class-wise metrics then provide recall (sensitivity), specificity, and precision for each class.

Class-wise performance metrics were calculated from the integrated confusion matrix. Sensitivity, specificity, and accuracy were 0.676, 0.640, and 0.719 for the slow-release class and 0.640, 0.676, and 0.593 for the fast-release class, respectively. Rather than specific predictive robustness, these numbers show class-wise performance that is modest and fairly balanced. Therefore, rather than being a verified tool for future formulation decision-making, the XGBoost model should be understood as an initial classifier that detects a statistically significant signal in the current dataset.

## 4. Discussion

Kinetic-model benchmarking using f₂ showed that apparent agreement between a fitted model and a liposomal release profile depends strongly on the selected model form. In this dataset, Weibull provided the highest overall similarity, while first-order and Korsmeyer–Peppas generally outperformed zero-order and Hixson–Crowell. Together, these comparisons suggest that many profiles are better described by non-linear model forms rather than strictly linear release behavior, consistent with multi-mechanism transport in liposomal systems. The PCA-k-means method was able to provide an interpretable clustering based on release-profile data which identified three clusters representing slow, intermediate and fast kinetic phenotypes having different average release-trajectories. By retaining the primary kinetic structure in the reduced-dimension standardized-release-vectors a sufficient number of release-profiles could be compared on a unified basis. Additionally, the resulting PCA-k-means embedding can be used for other similar operations (i.e., search for similarity, find outliers, etc.) and identify acceptable kinetic ranges especially when data from various IVR-conditions is combined.

Explainable Modeling showed that the IVR Temperature (the lipid mixture’s Transition Temperature) were the two most important variables for determining if a release was going to be fast or slow. Additional explanatory power came from drug loading and pH of the release media. This is consistent with the Mechanistic Model’s assertion that the observed release behavior is due to the membrane’s thermodynamic properties (thermodynamics), how the drug is distributed throughout the membrane, and the drug’s solubility/ionization state in the media. The fact that the SHAP values varied widely across samples highlights the heterogeneity of the sample data and the likelihood that there were non-linear interactions and possibly confounded relationships between the formulation design parameters and the experimental test conditions.

Permutation testing confirmed that XGBoost performed better than the other examined classifiers in terms of balanced accuracy, F1 score, and MCC, and that model performance was higher than would be predicted by chance within this dataset. The supervised sample size (n = 59) was adequate to show repeatable fold-to-fold performance trends, but it is still small and could add to the provided estimations’ uncertainty. Furthermore, modest assay-format or structural differences in infrequently represented categories may not be properly captured by integer encoding of categorical descriptors for tree-based models. The predictive signals found here should be viewed as correlative evidence that calls for future validation under controlled conditions, since formulation design factors and IVR conditions are generally expected to be related in the source literature. However, to increase the generalizability of the current findings, future research should include other lipid mixes, APIs, and IVR protocols to the database of release behavior. Mechanistic interpretation would be strengthened by adding mechanistically significant descriptors such buffer composition, sink-condition indicators, membrane composition fractions, and drug physicochemical features. In order to verify model generalizability and integrate the predictions into useful formulation and IVR method-development decisions, prospective validation on new formulation datasets produced under standardized conditions will ultimately be required.

## 5. Conclusion

A workflow for assessing heterogeneous liposomal IVR data that is integrated, explainable, and proof-of-concept is presented in this study. The method made it possible to compare models using f2 similarity and downstream PCA-k-means phenotyping by standardizing release profiles on a common time grid and parameterizing them with Weibull descriptors. A supervised XGBoost classifier had the best cross-validated performance for differentiating between slow and fast extremes after the investigation revealed slow, intermediate, and fast release phenotypes. However, the model should be considered preliminary and hypothesis-generating because the class-wise confusion-matrix findings show only moderate recall and precision. Media temperature, weighted lipid transition temperature, drug loading, and media pH were identified via feature selection and SHAP interpretation as significant descriptors that supported the workflow’s pharmacological plausibility. Overall, the results indicate that explainable machine learning might help with risk-based prioritization and the development of IVR methods for liposomal formulations. However, before the method can be applied for reliable prediction or regulatory decision-making, independent prospective validation using standardized experimental datasets is essential.
